# Complement activation capacity in plasma before and during high-dose prednisolone treatment and tapering in exacerbations of Crohn's disease and ulcerative colitis

**DOI:** 10.1186/1471-230X-5-31

**Published:** 2005-09-22

**Authors:** Erik Zimmermann-Nielsen, Henning Grønbæk, Jens Frederik Dahlerup, Gunnar Baatrup, Ole Thorlacius-Ussing

**Affiliations:** 1 Department of Surgery K, Hospital of Funen, Svendborg, Valdemarsgade 53, Denmark 5700 Svendborg; 2 Department of Medicine V, Aarhus University Hospital, Nørrebrogade 44, Denmark 8000 Aarhus C; 3 Department of Surgical Gastroenterology, Haukeland University Hospital, Norway Bergen; 4 Department of Surgical Gastroenterology A, Aalborg Hospital, Hobrovej 18-22, Postbox 365, Denmark 9100 Aalborg

## Abstract

**Background:**

Ulcerative colitis (UC) and Crohn's disease (CD) are characterized by intestinal inflammation mainly caused by a disturbance in the balance between cytokines and increased complement (C) activation. Our aim was to evaluate possible associations between C activation capacity and prednisolone treatment.

**Methods:**

Plasma from patients with exacerbations of UC (n = 18) or CD (n = 18) were collected before and during high dose prednisolone treatment (1 mg/kg body weight) and tapering. Friedman's two way analysis of variance, Mann-Whitney U test and Wilcoxon signed-rank sum test were used

**Results:**

Before treatment, plasma from CD patients showed significant elevations in all C-mediated analyses compared to the values obtained from 38 healthy controls (p < 0.02), and in mannan binding lectin (MBL)-concentration and MBL-C4-activation capacity (AC) values compared to UC patients (p < 0.02). Before treatment, plasma from UC patients showed significant elevations only in the classical pathway-mediated C3-AC compared to values obtained from healthy controls (p < 0.01). After treatment was initiated, significant reductions, which persisted during follow-up, were observed in the classical pathway-mediated C3-AC and MBL-C4-AC in plasma from CD patients (p < 0.05).

**Conclusion:**

Our findings indicate that C activation capacity is up-regulated significantly in plasma from CD patients. The decreases observed after prednisolone treatment reflect a general down-regulation in immune activation.

## Background

The complement (C) system consists of more than 20 proteins and a number of cell associated regulator molecules and receptors [1]. The C system is activated through either of three pathways, initiated by e.g. microorganisms, immune complexes (IC), and tissue injuries.

The classical pathway (CP) is initiated by IgM and IgG-molecules, bound to antigens, which trigger the activation of proenzymes leading to cleavage of C2 and C4, and eventually to cleavage of C3.

Cleavage of C3 is a key reaction in the C sequence as the classical and alternative pathways (AP) converge here, and from this point on potent anaphylactic and chemotactic C split-products are generated. Alternative pathway activation is initiated by CP-generated C3b or by a continuous low 'tick-over' activation of C3, generating C3b which binds randomly to available cell surfaces. Mannan-binding lectin (MBL) is the only known collectin known to activate the C system and binds multivalently to terminal mannose, N-acetylglucosamine, glucose and fucose on yeast cells and Gram-negative bacteria, thereby promoting phagocytosis of the microorganisms without the involvement of antibodies.

The MBL-pathway converges with the CP at the level of C4 which may be activated to generate C4b, which binds randomly to available surfaces. The activity of C, and especially C3b/iC3b and C4b/iC4b, is strictly regulated by proteins present ubiquitously in fluid-phases and expressed on almost all cell surfaces. Further insights into C continue to emerge, e.g. that MBL utilizes two specific serine proteases (MASP-1, MASP-2) to initiate the activation of C, and the function of these proteases may influence the pathogenesis of more diseases.

A role of the C system in the pathogenesis of ulcerative colitis (UC) and Crohn's disease (CD), or in maintaining inflammation, has been established by several observations: Mucosal cells show a down-regulation in their expression of C regulators within the affected areas of UC and CD [2]. A concomitant occurrence of IC and C activation has been demonstrated in plasma from UC and CD patients during clinical exacerbations [3-5]. Deposits of several C components in colonic mucosal are found to correlate significantly to the degree of inflammation in UC and CD [6,7]. A putative auto-antigen is demonstrable in colonic mucosal and in the extraintestinal tissues affected by UC [7,8], this auto-antigen may trigger C activation. The C split-product C5a may participate in the formation of the granulomas observed in colonic tissue affected by CD [9]. Thus, both AP and CP-mediated C activation have been suggested to be involved in UC and CD [10].

Glucocorticoids suppress inflammatory processes, e.g. by down-regulation of the transcription/translation of proinflammatory cytokines such as tumor necrosis factor alpha and interleukin-6 [11], presumeable by an inhibition of nuclear factor kappa B [12]. The effects of glucocorticoids on C activation have been reported inconsistently. This is partly explained by the dependency on the type and dosage of glucocorticoid administered, and the biphasic response in C activation with time after administration [13]. Glucocorticoids may enhance the synthesis of C1-inhibitor and interfere with the function of C3 convertases, thereby exerting an anti-complementary effect [14-16]. Glucocorticoids may inhibit C-mediated chemotaxis [17].

Measurement of C activation has been impeded by the complexity, lability and reactivity of the system. The methods currently in use for routine analyses show rather low sensitivity, and do normally not differentiate between the pathways, and the serum concentration of MBL varies considerably amongst individuals influenced by mutations and polymorphism in the promoter region [18]. We have standardized two functional assays which measure the C activation capacity of the classical, alternative and MBL pathways *in vitro *as function of incubation time. Our aim was to evaluate C in plasma before and during high-dose prednisolone treatment and tapering to analyze for a possible association between C activation capacity and treatment.

## Methods

Patients with clinical exacerbation of UC (n = 18) or CD (n = 18), necessitating hospitalization and requiring high-dose prednisolone to induce remission, were included consecutively at the Medical Department V, Aarhus University Hospital in the period December 2000 to July 2001. Patients were excluded by the presence of obvious bacterial infection or acute abdomen.

All patients had a well-established diagnosis of UC or CD according to clinical, biochemical, endoscopy, histopathological criteria, and small bowel follow through.

The group of patients with UC consisted of 6 women and 12 men, median 39 years of age (range 21–78). There were 12 women and 6 men among the patients with CD, median 29 years of age (range 18–78).

The disease activity was evaluated clinically by the Harvey-Bradshaw Index in both UC and CD patients [19]. The UC patients had a median score of 7 (range 5–19), and CD patients revealed a median score of 7 (range 3–24). Seventeen UC patients had extensive colitis (pancolitis) and 1 left sided colitis. Patients with CD had either colon affection, small bowel affection, or a combination.

At inclusion, UC patients were treated with combinations of steroids (rectal enema)(n = 6), 5-aminosalicylic acid (n = 12), antibiotics (n = 1), whereas 6 patients received no immunomodulatory medication at all. The CD patients were treated with combinations of steroids (rectal enema)(n = 1), 5-aminosalicylic acid (n = 7), azathioprine (n = 1), antibiotics (n = 1), whereas 4 patients received no immunomodulatory medication at all.

All treatments except for rectal steroid enema (except for 1 patient) and antibiotics were continued. The treatment with prednisolon was started instantly, and administrated intravenously for the first 3–7 days (1 mg/kg body-weight), followed by oral administration by day 7 where the dose were tapered to 40–60 mg/day, and further tapering typically 5 mg/week until 0.

Blood samples were collected from all patients before high-dose prednisolone treatment was initiated, after 7 days of high-dose prednisolone treatment, after 6 weeks when the prednisolone dosage was approximately 0.5 mg/kg, and after 12 weeks when prednisolone was stopped for most patients (except in 6 UC- and 4 CD patients). The patients still treated with prednisolon after 12 weeks were all well characterized by clinical and/or biochemical relapse during the last tapering period.

Plasma samples from healthy blood donors (n = 38) from the Department of Clinical Immunology, Aalborg Hospital were included as controls. In addition, plasma samples from some of these donors were heat inactivated at 56°C for 30 min and used as negative internal controls.

The blood samples were collected in citrate tubes by venepuncture without stasis, placed on ice for a maximum of 10 min, and centrifuged at 4°C and 2,000 g for 20 min. Duplicate blood samples were collected in glass tubes and allowed to clot at room temperature before centrifuged. Plasma/serum was withdrawn and stored in aliquots of 0.5 ml at minus 80°C.

Plasma/serum samples were analyzed for alternative- and classical C pathway mediated factor C3 activation capacity (C3-AC), mannan-binding lectin (MBL), MBL-C4-AC, leucocyte count, C-Reactive Protein (CRP) and orosomucoid.

The AC-derived measurements were performed at the Surgical Gastroenterological Research Unit whereas the MBL-assay was performed as a routine method at the Department of Clinical Immunology, Aalborg Hospital, as described in details elsewhere [20-22].

### C3-AC-assay

The enzyme linked immunosorbent assay (ELISA) measures plasma derived C3b/iC3b deposition on IC during *in vitro *C activation. The activation capacity of the C system is defined as the amount of C3b/iC3b generated and bound to the solid-phase IC as a function of incubation time. The assay differentiates between activation mediated by AP and CP. Briefly, the plasma was diluted 1/5 in MgEGTA-buffer (10 mM Mg^2+ ^and 10 mM EGTA) when measuring the AP and 1/200 in CaMg-buffer (0.30 mM Ca^2+ ^and 1.0 mM Mg^2+^) when measuring the CP. The AP is activated at low plasma dilutions only and the CP activation requires Ca^2+^. Upon C activation, the generated C3b molecules bind covalently to the IC and are eventually degraded to iC3b. Bound C3b/iC3b fragments were detected by the addition of biotinylated rabbit anti-C3c-antibodies (Dako, Glostrup, Denmark), avidin alkaline phosphatase, and para-nitrophenylphosphate as enzyme substrate. In standard dose response curves the absorbance values for plasma dilutions 1/5 (AP) and 1/200 (CP) were designated 100%. Plasma from 1 healthy donor was heat inactivated at 56°C for 30 min, and used as negative control. Test samples were analyzed in duplicate and the mean value was converted to 'per cent of the standard' [20].

A second ELISA measured the plasma concentration of MBL. Briefly, microplates were coated with anti-MBL-antibodies, plasma was diluted 1/100 in dilution-buffer (6.7 g NaCl, 4.6 g NaH_2_PO_4_·2H_2_O, 1.1 g KH_2_PO_4_, 5 mM EDTA, 200 μl mouse IgG). Bound MBL were detected by the addition of biotinylated anti-MBL-antibodies, avidin-peroxidase conjugate, and 1,2-phenylene diamine dihydrochloride as enzyme substrate. A plasma standard diluted 1/25-1/3,200 was included in all plates. Plasma from 3 healthy donors with high, moderate and low concentration of MBL were included as controls. Test samples were analyzed in duplicate and the mean value was used in the calculations [21].

The third ELISA measured the plasma derived C4b/iC4b of the MBL-pathway deposited on mannan during *in vitro *C activation. Briefly, microtiter plates were coated with mannan, and plasma was diluted 1/10 in diluent-buffer (10 mM tris hydroxy aminomethan, 10 mM CaCl_2_, 1 M NaCl, 15 mM NaN_3_, pH 7.8). The assay does not measure activation of the AP at the plasma dilution (1/10) used and the CP was not initiated due to the high NaCl-concentration in the diluent buffer (1 M). Bound C4b/iC4b fragments were detected by the addition of biotinylated rabbit anti-C4c-antibodies (Dako, Glostrup, Denmark), avidin alkaline phosphatase, and para-nitrophenylphosphate as enzyme substrate. A plasma standard diluted 1/5-1/100 was included. Test samples were analyzed in duplicate and the mean value was converted to 'per cent of the standard'. Plasma from 2 healthy donors with high and low MBL-C4-AC were included as controls. Plasma from 1 healthy donor was heat inactivated at 56°C for 30 min, and used as negative control [22].

The measurements of leucocyte count, CRP, and orosomucoid were performed by routine methods at the Department of Clinical Chemistry, Aarhus University Hospital.

### Ethics

This investigation has been approved by the regional Ethical Committees of Northern Jutland and Aarhus Counties, and wasin accordance with the standards of the Declaration of Helsinki II.

### Statistics

Non-parametric descriptive (median – range) and comparative statistics were used with a probality value of < 0.05 considered statistically significant. Data were analyzed by non-parametric methods. The calculations and analyses were performed with Prism 3.0 (GraphPad Software Inc., Microsoft Corp., USA). First, overall comparison over time (before treatment, 1 week, 6 weeks and 12 weeks after treatment) of a variable in the same group of patients (e.g. CD patients) was done by Friedman's two-way analysis. Secondly, paired comparison in a group of patients of a variable between two different time points (matched pair) were by Wilcoxon signed-rank test. Furthermore, the Mann-Whitney U test was used comparing one variable between two different groups at a specific time point (e.g. plasma value of MBL before prednisolone treatment in CD patients versus UC patients). Correlation between two variables were calculated using Spearman's rank correlation coefficient rho. Finally, to allow for multiple comparisons the Bonferroni correction was used, the corrected p-values are stated.

## Results

All C data showed a significant variation over time (before and 1, 6 and 12 weeks after initiation of high dose prednisolone treatment) in both UC and CD patients (in both groups: p < 0.0001, Friedman's test).

Table 1 shows the C-activation

### Before treatment

Plasma from CD patients revealed significant elevations in the activating capacity of all C pathways compared to the values obtained from healthy controls (p < 0.02, Mann-Whitney test). In plasma from UC patients, only the median classical pathway-mediated C3-AC was significantly elevated compared to the values obtained from healthy controls (p < 0.01, Mann-Whitney test). Plasma from CD patients revealed significant elevations in MBL-concentration and MBL-C4-AC values compared to the values obtained from UC patients (p < 0.02, Mann-Whitney test). No significant difference was observed in the alternative or classical pathway-mediated C3-AC between the patient groups.

### After treatment

No significant difference was observed neither in the alternative nor in the classical pathway-mediated C3-AC between the patient groups. The MBL-concentration and MBL-C4-AC values obtained were significantly higher at week 6 and 12 in plasma from CD patients compared to UC patients (p < 0.04, Mann-Whitney test).

### In CD patients

The classical pathway-mediated C3-AC and MBL-C4-AC remained significantly reduced over time in plasma from CD patients (p < 0.05, Wilcoxon test). In UC patients: No significant difference over time was observed.

### Other parameters

The medians of leucocyte count, CRP, and orosomucoid which were obtained from blood/plasma of CD and UC patients are stated in Table 2. Before treatment, plasma from CD patients revealed significant elevations in CRP and orosomucoid (p < 0.04, Mann-Whitney test). After treatment, the medians of all parameters were comparable.

The degrees of correlation were calculated for all C-values against CRP, orosomucoid, leucocyte count, and the Harvey-Bradshaw index, respectively. No signification correlation was observed

## Discussion

Our findings indicate that C activation capacity is up-regulated in plasma from CD patients compared to both healthy controls and UC patients, and that the decreases observed after treatment with prednisolone reflect a general down-regulation in immune activation as indicated by the concomitant trends in CRP and orosomucoid. The initial increase in leucocyte count may reflect a mobilization of premature leucocytes from the bone marrow. The demonstrated association between C activation capacity and treatment with prednisolone seems to support the finding that glucocorticoids may interfere with C activation capacity [13-16]. However, the concentrations of C components in plasma depend on a complex balance, e.g. between synthesis and degradation rates, and degree of binding to other proteins.

The C system has been suggested to be involved in the pathogeneses of UC and CD, although differently. Thus, a putative auto-antigen, with a potential to initiate the classical pathway, is demonstrable in tissues affected by UC [7,8], whereas C activation may occur through the alternative pathway in patients with CD [10]. The lectin-mediated C activation has not been considered in earlier studies of this topic.

More clinical features differ between UC and CD. Thus, UC is characterized by inflammatory infiltrations within the mucosa of the colon and rectum, whereas the infiltrations in CD may affect all layers of the intestinal wall. The clinical exacerbations, frequently necessitating hospitalization and glucocorticoid treatment, may vary widely between UC and CD, and by the duration of disease. These variations in clinical features may not be detectable with the Harvey-Bradshaw index.

Our results show more differences in plasma derived C activation capacity between patients with UC and CD, which is in concordance with the overall immune activation as indicated by CRP, orosomucoid and leucocyte count, and by the fact that CRP may activate C by initiating the classical pathway [23]. The up-regulation in C activation capacity is more pronounced in plasma from CD-patients compared to UC, in accordance with *in vitro *studies [24,25].

The demonstrated reduction of MBL-concentration and MBL-C4-AC in plasma from UC patients before treatment, compared to values obtained from either healthy controls or CD patients, is unexpected. Thus, the MBL gene variants, correlating with reduced MBL concentrations, occur less frequently in UC patients compared to CD patients and healthy controls [26]. However, our finding may be secondary to the use of immunomodulators at study entry, which was administered more frequently to the UC patients, or to differences in clinical features between UC and CD.

The significantly increased MBL-concentration and MBL-C4-AC values in plasma from CD patients, compared to values obtained from healthy controls, are opposite to our results in plasma from CD patients complicated by fistulizing ano-rectal disease [27] despite their comparability in disease activity as indicated by the Harvey-Bradshaw values. The discrepancy in MBL-concentration and MBL-C4-AC values of these two groups of CD-patients may be due to the circumstance that the patients with fistulizing disease were highly selected as chronic out-patients with a low degree of inflammation as indicated by CRP. In the present study only one of the CD patients had fistulizing ano-rectal disease.

The demonstrated association between C activation capacity and treatment with prednisolone seems to reflect a treatment-induced down-regulation in inflammation, as estimated by CRP and orosomucoid.

## Conclusion

We found that C activation capacity is up-regulated in plasma from CD patients compared to UC patients and healthy controls. The plasma MBL-concentration and MBL-mediated C activation capacity were reduced in UC patients compared to healthy controls. The changes in C activation capacity during prednisolone treatment seems to reflect a general down regulation in immune activation especially in CD patients.

## Competing interests

The author(s) declare that they have no competing interests.

## Authors' contributions

EZ-N participated to the conception and design of the study, carried out the immunoassays, participated to analysis and interpretation of data, and drafted the manuscript. HG participated to the conception and design of the study, collection of data, and helped to draft the manuscript. JFD participated to the design of the study, and was involved in revising the article critically. GB participated to the conception and design of the study, carried out the immunoassays, participated to analysis and interpretation of data, and drafted the manuscript. OTU participated to the conception and design of the study, to analysis and interpretation of data, and was involved in revising the article. All authors read and approved the final manuscript.

## Pre-publication history

The pre-publication history for this paper can be accessed here:



## Figures and Tables

**Table 1:** Complement activation in plasma from patients with Crohn's disease (CD), patients with ulcerative colitis (UC) and healthy subjects (HS) before and after 1, 6 and 12 weeks high-dose treatment with prednisolone.

	before treatment	after 1 week	after 6 weeks	after 12 weeks
	Number of patients	Number of patients	Number of patients	Number of patients
CD	18	18	18	18
UC	18	18	18	16
HS	38	--	--	--
**C3-AC, alternative**	% of standard	% of standard	% of standard	% of standard
CD	61.5 (53–82)	51.5 (49–71)	55.5 (49–70)	59 (49–75)
UC	58 (49–78)	49 (49–70)	49 (49–78)	59 (49–70)
HS	49 (49–67)	--	--	--
**C3-AC, classical**	% of standard	% of standard	% of standard	% of standard
CD	109 (81–172)	97.5 (39–124)	94.5 (70–141)	99.5 (53–143)
UC	97 (39–148)	88 (58–129)	91 (59–115)	94 (39–160)
HS	82.5 (68–108)	--	--	--
**MBL-concentration**	ng/ml	ng/ml	ng/ml	ng/ml
CD	3251 (14 – 6085)	3071 (10 – 4636)	2911 (10 – 5178)	3257 (10 – 4718)
UC	1082 (6 – 3926)	1171 (3 – 4845)	1141 (3 – 4590)	1292 (3 – 4147)
HS	1219 (2 – 3577)	--	--	--
**MBL-C4-AC**	% of standard	% of standard	% of standard	% of standard
CD	118 (21–187)	63 (25–192)	62.5 (21–163)	87 (17–192)
UC	54 (12–201)	54 (11–192)	45 (9–163)	55 (12–192)
HS	66.5 (9–162)	--	--	--

Median and range (in parenthesis) of alternative and classical pathway-mediated C3-activation capacity(C3-AC), mannan-binding lectin (MBL) concentration, MBL-C4-AC.

**Table 2:** Acute phase reactants (median values) in plasma/blood from patients with Crohn's disease (CD), patients with ulcerative colitis (UC) before and after 1,6 and 12 weeks high-dose treatement with prednisolone.

	before treatment	after 1 week	after 6 weeks	after 12 weeks
**C Reactive Protein**				
(nmol/L)				
CD	372	47	47	62.5
UC	47	47	47	33.5
**Orosomucoid**				
(μmol/L)				
CD	39	33	28	25
UC	25	25	20	24
**Leucocyte count**				
(10^9^/L)				
CD	10.9	13.5	9.7	8.1
UC	8.3	13.6	8.6	7.9
